# Complications of fixed infrared emitters in computer-assisted total knee arthroplasties

**DOI:** 10.1186/1471-2474-8-71

**Published:** 2007-07-27

**Authors:** Daniel Hernández-Vaquero, Abelardo Suárez-Vázquez

**Affiliations:** 1 Department of Orthopaedic Surgery, Hospital San Agustin, Avilés School of Medicine, University of Oviedo, Spain

## Abstract

**Background:**

The first stage in the implant of a total knee arthroplasty with computer-assisted surgery is to fasten the emitters to the femur and the tibia. These trackers must be hard-fixed to the bone. The objectives of our study are to evaluate the technical problems and complications of these tracker-pins, the necessary time to fix them to the bone and the possible advantages of a new femoral-fixed tracker-pin.

**Methods:**

Three hundred and sixty seven tracker-pins were used in one hundred and fifty one computer-assisted total knee replacements. A bicortical screw was used to fix the tracker to the tibia in all cases; in the femur, however, a bicortical tracker was used in 112 cases, while a new device (OrthoLock) with percutaneous fixation pins was employed in the remaining 39.

**Results:**

Technical problems related to the fixing of the trackers appeared in nine cases (2.5%). The mean surgery time to fix the tracker pin to the tibia was 3 minutes (range 2–7), and 5 minutes in the case of the femoral pin (range: 4–11), although with the new tool it was only three minutes (range 2–4) (p < 0.001). No complications were observed with this new device.

**Conclusion:**

The incidence of problems and complications with the fixing systems used in knee navigation is very small. The use of a new device with percutaneous pins facilitates the fixing of femoral trackers and decreases the time needed to place them.

## Background

Several studies have confirmed that the optimal positioning and alignment of prosthetic components are crucial for the best long-term outcomes. With the standard mechanical instrumentation, however, this is not possible in all cases [1]. Computer-Assisted Surgery (CAS) has shown its usefulness to achieve a better placement of Total Knee Arthroplasty (TKA) in the sagittal and coronal planes. Comparative studies between surgery with and without navigation assistance have demonstrated the advantages of CAS when it comes to bone cutting following the limb's mechanical axis. Although the long-term follow-up is still unknown, the improved placement of the implants will presumably offer better results in the TKA in the medium- and long-term [2].

There are different navigation systems in TKA; some need to previously acquire an image (CT-based systems) while others do not; some utilize wires to connect the trackers to the computer and others are wireless. Current imageless navigation systems consist of a workstation, an infrared optical camera system, and battery-powered wireless trackers. These trackers are rigidly fixed to the bone so that their position, when compared with the anatomically selected points, remains constant and any movement of the bone and its associated tracker position is recorded. The navigational tools rely on the communication between the light-emitting diodes on the trackers and the infrared camera that locates the tracker position in space. The positional information of the trackers, the attached bone, and the instruments are analyzed by the computer, which determines the real-time position of the leg within a 3-dimensional coordinate system. In the first models, three emitters fixed to the anterior superior iliac spine, distal femur and proximal tibia with bicortical screws were necessary. In the new systems, however, only two trackers, fixed to the distal femur and proximal tibia, are necessary. The objectives of our study are to evaluate the problems and complications which may arise while using these tracker-pins. We analyze the problems found with the placement of the trackers, any fixing flaws that forced the termination of the procedure and the complications related to the fixing of the trackers. We also studied the eventual advantages of a new femoral fixed emitter as to the necessary time for its fixation and as to the technical problems found.

## Methods

**Figure 1:**
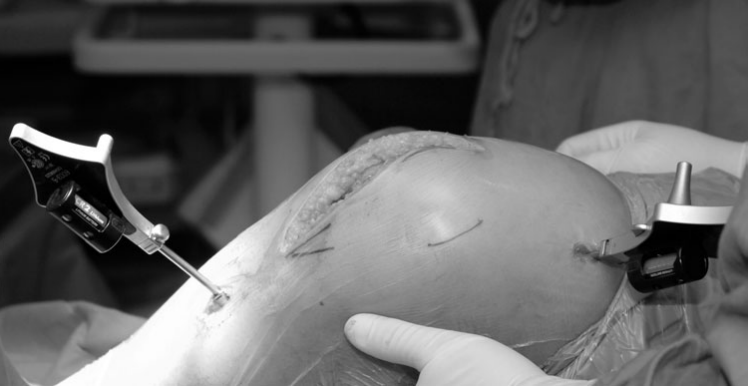
Total knee replacement with minimally invasive surgery and navigation.

One hundred and fifty one TKA were performed with navigation using an imageless, wireless system (Stryker-Leibinger, Freiburg, Germany) between January 2002 and March 2006. The last thirty two cases were operated using minimally invasive surgery and a midvastus approach (Figure 1). The study has been performed following the ethical recommendations of the Carlos III Health Institute (FIS 02/0226, FIS 05/1065), the Spanish National Healthcare System (Ministry of Health and Food) and it was approved by the Regional Ethic Committee. A specific informed consent was obtained from the patients included in the study.

**Figure 2:**
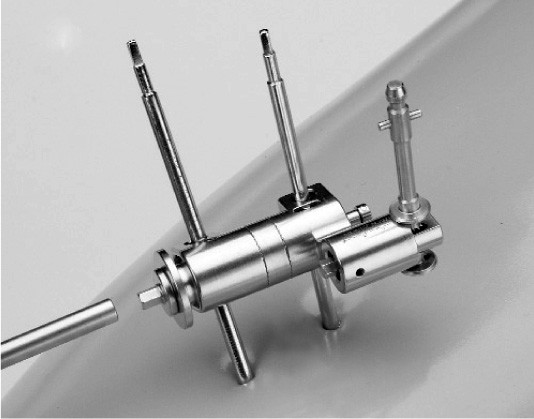
New femoral tracker fixing device.

Three hundred and sixty seven tracker-pins were used in total. In the first 65 TKR, an iliac crest screw was placed (software versions 1.2 and 1.3), but in the remaining patients it was unnecessary thanks to the use of a new software (versions 2.0 and 3.1). A bicortical screw (4 mm in diameter and a length of 20 – 60 mm) was used to fix the tracker pin to the tibia in all cases, whereas in the femur, a bicortical tracker was used in 112 cases and a new device with two percutaneous fixation pins (OrthoLock Anchoring Device) (Stryker-Leibinger) (3 mm in diameter × 150 mm, predrilled) (Figure 2) was used in the remaining 39. The tracker was mounted and tightly closed onto the tracker interface, which was clamped with a screwdriver. The stability of the tracker was checked manually by two surgeons. Anova test was used to compare the mean surgery time to fix the tracker pin to the femur between the bicortical tracker group and the OrthoLock group.

## Results

Technical problems arose in nine cases (2.5%) related to the fixing of the trackers. In three cases, the fixing of the iliac crest screw couldn't be performed and the navigation was aborted. In another three cases, the loosening of the femoral screw impeded the navigation and also was aborted. In two cases two attempts were necessary, and yet in another case three attempts were necessary (always with the femoral tracker). No complication was found when the OrthoLock device was used. The mean surgery time to fix the tracker pin to the tibia was 3 minutes (range 2–7) and 5 minutes for the femoral pins (range 4–11) (SD: 1.16). Fixation to the femur was reduced to 3 minutes (range 2–4) (SD: 0.33) with the new percutaneous OrthoLock device showing statistical significance (p < 0.001) compared with the bicortical group.

We didn't find any major complications such as fractures, infections or vascular- nervous damages. In three cases we found a painful scar in the tibial tracker hole that disappeared after two months.

## Discussion

CAS helps to improve the limb alignment of the TKA. Furthermore, it also seems to diminish the presence of systemic emboli [3] and to lessen the risk of transfusion [4,5]. Other authors have found that navigation-assisted patients showed statistically significant better results in the flexion range, HSS score, and WOMAC scores, when compared with conventional surgery patients [6]. A recent meta-analysis confirms that CAS improves the leg axis, although the numbers used in many of these studies were far too small to discriminate any other results.

The disadvantages related to the CAS are a longer surgery time and the possibility of complications and adverse effects due to tracker fixing. During the initial use of the navigation system, the placement of the trackers and the performance of the anatomic survey added approximately 15 to 20 minutes. Once the surgeon became familiar with these methods, the placement of the trackers and the anatomic survey added approximately 10–15 minutes [5,7].

There were a few complications related either to the computer software or the tracker pins inserted in the bones [8]. The placement of the pins and screws in the bone is inevitably associated with some risk. Using pins for tracker fixation creates potential damage issues to neurovascular structures and may cause the breakage of the devices inside the bone, although we did not experience any of these problems in our study. Another issue related to the pins required for fixation of the trackers was the use of a pin in the iliac crest with a separately prepared and draped area. Although there had been no known problems with this technique, it has been abandoned, since it is no longer necessary for the procedure thanks to the updated software. When the iliac crest tracker was used, Sikorski and Blythe [8] found a 6% of injuries to the lateral cutaneous nerve of the thigh with some persistent numbness, three pin-tract infections (3%), and two periprosthetic fractures, both of which occurred to elderly, osteoporotic patients and after major falls. One was through the site of the screw (the teeth produced an 'apple-corer' effect and a fracture of the shaft of the femur was associated with its use) and the other through a medial femoral condyle well away from the site of the screw. These authors also report a 4% of pin-tract infections in the tibia. Specific navigation complications were observed in two cases of the study undergone by Jenny et al [9]: one broken pelvic drill and one femoral screw forgotten; the authors, however, did not consider that these complications had any significant adverse effects. Three (5%) complications were related to the navigation process in another study [10]; in these three cases, rigid bodies were contaminated with blood, which led to tracking problems with the navigation system's camera. After cleaning the rigid bodies, the operations could be finished without any further problems.

A potential source of error can be a poor fixation of the locator to the bone, especially in the case of porotic bone. The accuracy of the system is totally dependent on the stability of the beacon or reflector arrays. Any movement altered the results. We consider that the tracker was firm enough when no movement was possible with the strength of one's hands although we admit that it is a subjective finding.

Throughout the years, bone fixation systems have been modified to allow for a more secure fixation. The new OrthoLock system allows for a faster fixation, a percutaneous placement and a more versatile positioning of the femoral emitters.

## Conclusion

The incidence of problems and complications of the fixing systems used in navigation is very small. The time needed to place them is close to six minutes. Thus, the use of a new device with percutaneous pins facilitates the fixing of femoral trackers.

## Competing interests

The author(s) declare that they have no competing interests.

## Authors' contributions

DHV conceived the study, participated in its design and coordination and drafted the manuscript.

ASV carried out the acquisition of data and participated in the design.

## Pre-publication history

The pre-publication history for this paper can be accessed here:


